# A Methodology to Model the Rain and Fog Effect on the Performance of Automotive LiDAR Sensors

**DOI:** 10.3390/s23156891

**Published:** 2023-08-03

**Authors:** Arsalan Haider, Marcell Pigniczki, Shotaro Koyama, Michael H. Köhler, Lukas Haas, Maximilian Fink, Michael Schardt, Koji Nagase, Thomas Zeh, Abdulkadir Eryildirim, Tim Poguntke, Hideo Inoue, Martin Jakobi, Alexander W. Koch

**Affiliations:** 1 Institute for Driver Assistance Systems and Connected Mobility (IFM), Kempten University of Applied Sciences, Junkersstrasse 1A, 87734 Benningen, Germany; 2 Institute for Measurement Systems and Sensor Technology, Technical University of Munich, Theresienstrasse 90, 80333 Munich, Germany; 3 Advanced Vehicle Research Institute, Kanagawa Institute of Technology, Shimoogino 1030, Atsugi 243-0292, Japan; 4 Blickfeld GmbH, Barthstrasse 12, 80339 Munich, Germany; 5 Infineon Technologies Austria AG, 4040 Linz, Austria

**Keywords:** LiDAR sensor, rain, fog, sunlight, advanced driver-assistance system, backscattering, Mie theory, open simulation interface, functional mock-up interface, functional mock-up unit

## Abstract

In this work, we introduce a novel approach to model the rain and fog effect on the light detection and ranging (LiDAR) sensor performance for the simulation-based testing of LiDAR systems. The proposed methodology allows for the simulation of the rain and fog effect using the rigorous applications of the Mie scattering theory on the time domain for transient and point cloud levels for spatial analyses. The time domain analysis permits us to benchmark the virtual LiDAR signal attenuation and signal-to-noise ratio (SNR) caused by rain and fog droplets. In addition, the detection rate (DR), false detection rate (FDR), and distance error $d_{error}$ of the virtual LiDAR sensor due to rain and fog droplets are evaluated on the point cloud level. The mean absolute percentage error (MAPE) is used to quantify the simulation and real measurement results on the time domain and point cloud levels for the rain and fog droplets. The results of the simulation and real measurements match well on the time domain and point cloud levels if the simulated and real rain distributions are the same. The real and virtual LiDAR sensor performance degrades more under the influence of fog droplets than in rain.

## 1. Introduction

Highly automated vehicles perceive their surroundings using environmental perception sensors, such as light detection and ranging (LiDAR), radio detection and ranging (RADAR), cameras, and ultrasonic sensors. LiDAR sensors have gained significant attention over the past few years for their use in advanced driver-assistance system (ADAS) applications because they provide outstanding angular resolution and higher-ranging accuracy [1]. As a result, the automotive LiDAR sensor market is predicted to be worth around 2 billion USD in 2027, up from 26 million USD in 2020 [2]. However, it is common knowledge that the LiDAR sensor performance degrades significantly under the influence of certain environmental conditions, such as rain, fog, snow, and sunlight. The effects of these weather phenomena must therefore be taken into account when designing LiDAR sensors, and the measurement performance of the sensors must be validated before using them in highly-automated driving settings. Furthermore, billions of miles of test driving are required for automated vehicles to demonstrate that they reliably prevent fatalities and injuries, which is not feasible in the real world due to cost and time constraints [3]. Simulation-based testing can be an alternative to tackle this challenge, but it requires that the effects of rain and fog are modeled with a high degree of realism in the LiDAR sensor models so they can exhibit the complexity and the behavior of real-life sensors.

In this work, we have modeled the rain and fog effect using the Mie scattering theory in the virtual LiDAR sensor developed by the authors in their previous work [4]. It should be noted that the sensor model considers scan pattern modeling and the real sensor’s complete signal processing. Moreover, it also considers the optical losses, inherent detector effects, effects generated by the electrical amplification, and noise produced by sunlight to generate a realistic output. The sensor model is developed using the standardized open simulation interface (OSI) and functional mock-up interfaces (FMI) to make it tool-independent [4]. We have conducted the rain measurements at the large-scale rain area of the National Research Institute for Earth Science and Disaster Prevention (NIED) in Japan [5] and the fog measurements at CARISSMA (Technische Hochschule Ingolstadt, Germany) [6]. The real measurements and the simulation results are compared to validate the modeling of the rain and fog effects on the time domain and point cloud levels. Furthermore, key performance indicators (KPIs) are defined to validate the rain and fog effects modeling at the time domain and point cloud levels.

The paper is structured as follows. Section 2 describes the LiDAR sensor background, followed by an overview of the related work in Section 3. The modeling of rain and fog effects is described in Section 4, and the results are discussed in Section 5. Finally, Section 6 and Section 7 provide the conclusion and outlook.

## 2. Working Principle

**Figure 1:**
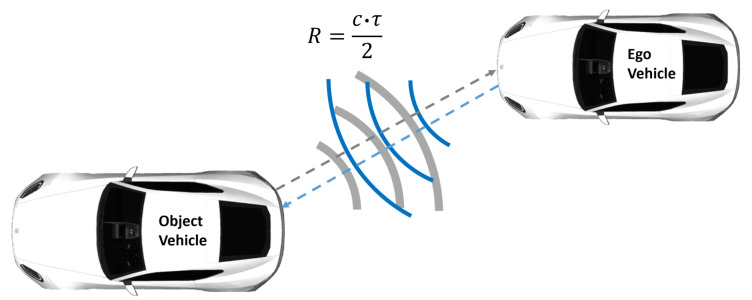
LiDAR working principle. The LiDAR sensor mounted on the ego vehicle simultaneously sends and receives laser light, which is partly reflected off the surface of the target, in order to measure the distance [4].

LiDAR is an optical technology that obtains distance information by bouncing light off an object. LiDAR sensors measure the round-trip delay time (RTDT) that laser light takes to hit an object and bounce back to the receiver to calculate the range, as shown in Figure 1.

Mathematically, the measured range *R* can be written as:

(1) $$R=\frac{c\;\cdot \;\tau }{2},$$

where *c* is the speed of light and RTDT is denoted by τ.

## 3. Related Work and Introduction of a Novel Approach

The influence of rain and fog on the LiDAR signal is well-described in the literature. For example, Goodin et al. [7] proposed a mathematical model to predict the influence of rain on the received power and range reduction. Afterward, they investigated the impact of the LiDAR performance degradation caused by rain on an obstacle-detection algorithm. Wojtanowski et al. [8] described the signal attenuation and decrease in the maximum detection range of LiDAR sensors operating both at 905 nm and 1500 nm due to the effects of rain and fog. In addition, they have shown that the LiDAR sensors at 905 nm are less sensitive to environmental conditions than the ones at 1500 nm. Rasshofer et al. [9] used the Mie scattering theory to model the influence of the effect of rain, fog, and snow on the performance of LiDAR sensors. Their signal attenuation model results show good agreement with the real measurements, but they do not quantify the attenuation of the LiDAR signals. Furthermore, they show a reduction in the maximum detection range of LiDAR sensors once the rain rate increases up to 18 mm/h. Byeon et al. [10] also modeled the LiDAR signal attenuation caused by raindrops using the Mie scattering theory. They used the rain distribution of three regions to show that the LiDAR signal attenuation varies according to rain distribution characteristics. The LiDAR sensor model by Li et al. [11] considers the LiDAR signal attenuation caused by rain, fog, snow, and haze. Zhao et al. [12] extended the work of [11] and modeled the unwanted raw detections (false positives) due to raindrops. They verified their model intuitively by comparing it with the data obtained during uncontrolled outdoor measurements. In Ref. [13], the authors have developed a model to predict and quantify the LiDAR signal attenuation due to raindrops. The simulation results matched well with the real measurements, with a deviation of less than 7.5%. Hasirlioglu et al. [14,15] introduced a noise model that adds unwanted raw detections (false positives) to the real LiDAR data based on the hit ratio. The hit ratio determines whether the laser beam hits the raindrop or not. If the hit ratio value exceeds the selected threshold, a false positive scan point will be added to the real point clouds. One drawback, however, of this approach is that it is computationally expensive. They verified their modeling approach by comparing real point clouds modified by the noise models with the points obtained under rainy conditions. The intensity values of the false positive scan points are set empirically because no internal information about the LiDAR hardware is available. Berk et al. [16] proposed a probabilistic extension of the LiDAR equation to quantify the unwanted raw detection by LiDAR sensors due to raindrops. They combined the probabilistic models’ parameters for rain distribution with the Mie scattering theory and the LiDAR detection theory in the Monte Carlo Simulation. However, they did not verify their model by comparing it with real measurements. In Ref. [17], the authors proposed a real-time LiDAR sensor model that considers the beam propagation characteristics and rain noise based on a probabilistic rain model developed in [16]. Their sensor model uses an unreal engine’s ray casting module, providing the LiDAR point clouds. The sensor model is not validated by the measurements of the real LiDAR sensor. Kilic et al. [18] introduced a physics-based simulation approach to add the effect of rain, fog, and snow on the real point clouds obtained under normal weather conditions. Their results show that the LiDAR-based detector performance improved once they trained with the LiDAR data obtained under normal and adverse weather conditions. Hahner et al. [19] simulated the effect of fog on the real LiDAR sensor point clouds and used the obtained foggy data to train the LiDAR-based detector to improve its detection accuracy.

All the state-of-the-art works mentioned above primarily focus on modeling the LiDAR signal attenuation and stochastic simulation of backscattering (false positives) caused by rain and fog droplets. However, most authors did not quantify these errors or validate their modeling approach by comparing the simulations with the real measurements. Some authors obtained the real LiDAR point clouds under normal environmental conditions and stochastically implemented a false positive effect caused by rain, fog, and snow. These approaches are helpful for use cases where the data from rain and fog are required for ADAS testing, regardless of their accuracy; however, they do not fit very well in the use cases where a high-fidelity virtual LiDAR sensor output is needed under adverse rain and fog conditions because the actual LiDAR sensor’s inherent optical detector and peak detection algorithm behavior significantly change depending on the rain intensity and fog visibility distance *V*. For instance, the LiDAR rays that are backscattered from the rain or fog droplets also increase the background noise of the detector, and the low-reflective targets close to the LiDAR sensor may be masked under the noise. This effect produces a false negative result, significantly decreasing the LiDAR sensor detection rate. Therefore, it is not the case that every backscattered LiDAR ray from the rain or fog droplets appears as a false positive scan point in the point cloud. Moreover, the scattering from the rain and fog droplets misaligns the LiDAR rays, shifts the target peak location, and ultimately results in the ranging error $d_{error}$. An overview of the features, output, and validation approaches of the state-of-the-art works mentioned above are tabulated in Table 1.

In this work, we introduce a novel approach to model the effect of rain and fog on the performance of the LiDAR sensor. The proposed methodology allows for the simulation of the rain and fog effect using the rigorous applications of the Mie scattering theory on the time domain for transient and point cloud levels for spatial analyses. This methodology can be used to analyze the behavior of LiDAR detectors and peak detection algorithms under adverse rain and fog conditions. We have compared the simulation and real measurements on the time domain to quantify LiDAR signal attenuation and signal-to-noise ratio (SNR) caused by rain and fog droplets. The detection rate (DR), false detection rate (FDR), and ranging error $d_{error}$ due to rain and fog droplets are evaluated on the point cloud level. In addition, we have generated a virtual rain and fog model that considers the drop size distribution (DSD), falling velocity, gravity, drag forces, turbulent flow, and droplet deformation.

## 4. Modeling of the Rain and Fog Effect in the Virtual LiDAR Sensor

**Figure 2:**
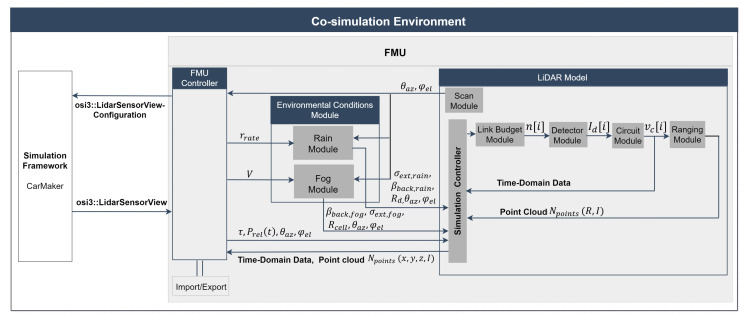
Co-simulation framework of the proposed approach to model the rain and fog effect in a virtual LiDAR sensor.

Figure 2 depicts the toolchain of the proposed approach and the signal processing steps to model the rain and fog effect in the virtual LiDAR sensor. As mentioned earlier in Section 1, the model is built using the standardized interfaces OSI and FMI and integrated into the virtual environment of CarMaker. This provides the ray tracing framework with a bidirectional reflectance distribution function (BRDF) that considers the direction of the incident ray θ, material surface, and color properties [20]. The LiDAR model uses the ray tracing module of CarMaker. The material properties of the simulated objects, the angle-dependent spectral reflectance $R_{\lambda }\left(\theta \right)$, and the reflection types (including diffuse, specular, retroreflective, and transmissive) are specified in the material library of CarMaker.

The FMU controller passes the required input configuration to the simulation framework via *osi3::LidarSensorViewConfiguration*. The simulation tool verifies the input configuration and provides the ray tracing detections via *osi3::LidarSensorView::reflection*, interface time delay τ, and relative power $P_{rel}\left(t\right)$ [4].

The FMU controller then calls the LiDAR model and passes the time delay τ, relative power $P_{rel}\left(t\right)$, azimuth $\theta_{az}$, and elevation $\varphi_{el}$ angles of the detected ray for further processing. In the next step, the FMU controller calls the environmental condition module and passes the user-selected rain rate ($r_{rate}$) or visibility distance *V* to the rain or fog modules. In the rain/fog module, virtual rain and fog are created, through which the scan module casts the LiDAR rays according to its scan pattern ($\theta_{az}$, $\varphi_{el}$), and a collision detection algorithm is applied to determine whether the transmitted ray of each scan point hits the rain or fog droplet; if the beam hits the droplet, the backscattered coefficient $\beta_{back}$ and the extinction coefficient $\sigma_{ext}$ based on the DSD are calculated. Furthermore, it also provides the spherical coordinates ($R_{d}$, $\theta_{az}$, $\varphi_{el}$) of the rain or fog droplets ($R_{cell}$, $\theta_{az}$, $\varphi_{el}$) that collided with the LiDAR ray. Next, the rain/fog module calls the LiDAR model and passes the rain or fog droplets data for further processing. The central component of the LiDAR model is the simulation controller. It is used as the primary interface component to provide interactions with the different components of the model, for instance, configuring the simulation pipeline, inserting ray tracing and the rain/fog module data, executing each step of simulation, and retrieving the results.

The link budget module calculates the arrival of photons over time. The detector module’s task is to capture these photons’ arrivals n[i] and convert them into a photocurrent signal $I_{d}\left[i\right]$. In this work, we have implemented silicon photomultipliers (SiPM) as a detector. Next, the circuit module amplifies and converts the detector’s photocurrent signal $i_{d}\left[i\right]$ to a voltage signal $v_{c}\left[i\right]$ processed by the ranging module. The last part of the toolchain is the ranging module, which determines the range *R* and intensity *I* of the target based on the $v_{c}\left[i\right]$ received from the analog circuit for every reflected scan point. The LiDAR point cloud $N_{points}$ is exported in Cartesian coordinates.

### 4.1. Scan Module

**Figure 3:**
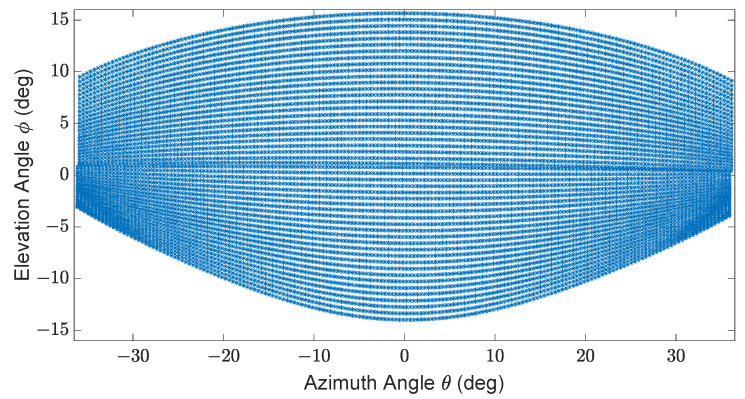
Exemplary scan pattern of Cube 1. ±36° horizontal and ±15° vertical FoV, 50 scan lines, 0.4° horizontal angle spacing, frame rate 5.4 Hz, maximum detection range 250 m, and minimum detection range 1.5 m.

The scan module uses the scan pattern of the Blickfeld Cube 1, as shown in Figure 3. A detailed description of the Blickfeld Cube 1 scan pattern can be found in [21].

### 4.2. Rain Module

The rain module generates virtual rain and calculates the extinction coefficient $\sigma_{ext,rain}$ and backscattered coefficient $\beta_{back,rain}$.

#### Virtual Rain Generation

Virtual rain is generated using the Monte Carlo Simulation. Monte Carlo Simulation uses a Mersenne Twister 19937 pseudo-random generator [22] to sample the given DSD using the inverse transform sampling method [23]. Each raindrop is generated individually based on an underlying DSD and terminal velocity. A similar approach was introduced by Zhao et al. [12] to generate unwanted raw data within the sensor models. DSD is the distribution of the number of raindrops by their diameter. Many types of DSD are described in the literature, including the Marshall–Palmer distribution, Gamma distribution, and Lognormal distribution [24,25,26]. In this work, we have used the Marshall–Palmer rain distribution and the large-scale rainfall simulator distribution recorded by a real disdrometer.

### 4.3. Marshall–Palmer Distribution

The Marshall–Palmer DSD is commonly used in the literature for characterizing rain, and it can be written as:

(2) $$N\left(D\right)=N_{0}\;\cdot \;e^{-\Lambda D},$$

where $N_{0}$ = 8000 m${}^{-3}$ mm${}^{-1}$, Λ = 4.1 $r_{rate}$${}^{-0.21}$ mm${}^{-1}$, $r_{rate}$ is the rain rate in mm/ h, and *D* is the drop diameter in mm [24].

We use the inverse transform sampling method [23] to randomly generate raindrops based on the Marshall–Palmer distribution. Equation (2) becomes:

(3) $$D=-\frac{1}{\Lambda }\;\cdot \;\ln\left(1-u\right),$$

where *u* is a random variable with the uniform distribution U(0,1) [23]. The terminal velocity $v_{t}$ of the raindrops in m/s can be written as:

(4) $$v_{t}\left(D\right)=3.78\;D^{0.67},$$

where *D* is the raindrop diameter in mm [27].

### 4.4. Physical Rain Model

**Figure 4:**
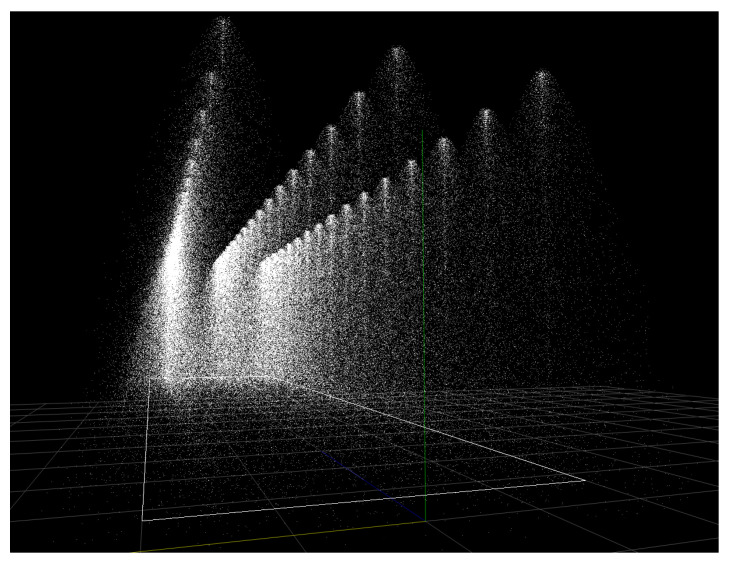
Exemplary visualization of a rain field generated by sprinklers resembling a real-world rain simulator.

The physical rain model aims to simulate the characteristics of a rain simulator inside the virtual simulation environment. In this work, we have generated the virtual rain according to the rain distribution of the NIED rain facility recorded by the real disdrometer. Rather than evenly distributing the droplets in the environment, they are “spawned” from rain sources, such as sprinklers or a homogenous planar rain source. Once the droplets are spawned, their movement is determined by physical equations concerning gravity, drag forces, turbulent flow, and droplet deformation according to the base concept published by John H. Van Boxel [28]. Figure 4 shows an exemplary visualization of the rain field generated with the physical rain model.

It is not within the scope of this paper to explain the detailed working principle of the physical rain model, nor will its mathematical model be discussed here. The physical rain model provides access to all the properties of the droplets, including position, speed, and diameter. Based on these properties and the characteristics of the sensors’ beam, the backscattered $\beta_{back,rain}$ and extinction $\sigma_{ext,rain}$ effects can be applied to the virtual LiDAR signal.

### 4.5. Interaction Between the Electromagnetic Waves and Hydrometeors

There are three scattering theories (Rayleigh, Mie, and geometric/ray optics) that can explain the interaction between the electromagnetic wave and hydrometeors depending on the size parameter $x=\frac{\pi \;D}{\lambda }$. When the particle size *x* is very small compared to the incident wavelength (x<<1), the Rayleigh scattering theory is used. Mie scattering theory is used when the incident wavelength and particle size are comparable or equal (x≈1). Geometric/ray optics is used when the incident wavelength is very small compared to particles (x>>1). The average raindrop diameter *D* is 1 mm, while the average fog droplet diameter *D* is 10 μm. The size parameter of the LiDAR sensor operating at a wavelength of 905 nm is x=3471 for raindrops and x=35 for fog droplets [29].

In conclusion, the interaction between LiDAR waves and raindrops can be explained using geometric/ray optics and the Mie scattering theory in the case of fog droplets. In Ref. [30], the authors have found that the results from the Mie scattering theory and geometric/ray optics agree well with each other for x≥400. We, therefore, use the Mie scattering theory to model the rain and fog effect on the LiDAR sensor performance.

#### Mie Scattering Theory

Scattering is the process where a particle changes the direction of the incident wave, while absorption is the process where heat energy is produced. Both scattering and absorption extract energy from the incident wave and are called extinction [31]. Mathematically, it can be written as:

(5) $$Q_{ext}=Q_{sca}+Q_{abs},$$

where $Q_{ext}$ is the extinction efficiency, $Q_{sca}$ denotes the scattering efficiency, and $Q_{abs}$ shows the absorption efficiency [31]. In addition, the raindrops backscattered the incident LiDAR ray, and this phenomenon is known as backscattered efficiency $Q_{back}$ [31].

The Mie scattering theory can be used to calculate the extinction efficiency $Q_{ext}$, the scattering efficiency $Q_{sca}$, and the backscattered efficiency $Q_{back}$ for LiDAR sensors. They can be calculated as:

(6) $$Q_{ext}=\frac{2}{x^{2}}\sum_{n=1}^{N_{max}}\left(2n+1\right)\;\cdot \;ℜ\left(a_{n}+b_{n}\right),$$

(7) $$Q_{sca}=\frac{2}{x^{2}}\sum_{n=1}^{N_{max}}\left(2n+1\right)\;\cdot \;\left(|a_{n}{|}^{2}+{\left|b_{n}\right|}^{2}\right),$$

(8) $$Q_{back}=\frac{1}{x^{2}}{\left|\sum_{n=1}^{N_{max}}\left(2n+1\right){\left(-1\right)}^{n}\;\cdot \;\left(a_{n}-b_{n}\right)\right|}^{2},$$

where *x* is the size parameter, *ℜ* denotes the real part, $N_{max}$ = $x+4x^{\frac{1}{3}}+10$, and $a_{n}$ and $b_{n}$ are the complex Mie coefficients [31]. The Mie coefficients can be calculated using the spherical Bessel functions [32] and the method introduced by Hong Du [33]. The Hong Du method is computationally less expensive, so we have used it in this work. According to this algorithm, the Mie coefficients can be written as:

(9) $$a_{n}=\frac{\left[r_{n}\left(mx\right)/m+n\left(1-1/m^{2}\right)/x\right]\Psi_{n}\left(x\right)-\Psi_{n-1}\left(x\right)}{\left[r_{n}\left(mx\right)/m+n\left(1-1/m^{2}\right)/x\right]\zeta_{n}\left(x\right)-\zeta_{n-1}\left(x\right)},$$

and:

(10) $$b_{n}=\frac{r_{n}\left(mx\right)m\Psi_{n}\left(x\right)-\Psi_{n-1}\left(x\right)}{r_{n}\left(mx\right)m\zeta_{n}\left(x\right)-\zeta_{n-1}\left(x\right)},$$

where *m* is the complex refractive index of water, the size parameter is denoted by *x*, $r_{n}\left(mx\right)=\frac{\Psi_{n-1}\left(mx\right)}{\Psi_{n}\left(mx\right)}$ presents the complex ratio of the Riccati–Bessel functions, and $\Psi_{n}\left(x\right)$ and $\zeta_{n}\left(x\right)$ are Riccati–Bessel functions. The complex ratio $r_{n}\left(mx\right)$ can be calculated using the upward and downward recurrences, and it can be written as:

(11) $$r_{n+1}\left(mx\right)={\left[\frac{2n+1}{mx}-r_{n}\left(mx\right)\right]}^{-1}.$$

The downwards recurrence of $r_{n}\left(mx\right)$ is initialized by:

(12) $$r_{N^{*}}\left(mx\right)=\left(2N^{*}+1\right)/\left(mx\right),$$

and the values are iteratively calculated until n=1. $N^{*}$ controls the precision of the Mie scattering coefficients, and it should be greater than $N^{*}>11,010$ if six significant digits or higher precision are required [33]; in this work, we have used $N^{*}>30,000$ to obtain seven significant digits precision. The Riccati–Bessel functions $\Psi_{n}\left(x\right)$ and $\zeta_{n}\left(x\right)$ of Equation (9) can also be calculated by using the upward and downward recurrences and can be written as:

(13) $$\Psi_{n+1}\left(x\right)=\left(2n+1\right)\Psi_{n}\left(x\right)/x-\Psi_{n-1}\left(x\right).$$

The complex function $\zeta_{n}\left(x\right)$ can be defined as:

(14) $$\zeta_{n}\left(x\right)=\Psi_{n}\left(x\right)+i\chi_{n}\left(x\right).$$

The same Formula (13) applies to $\chi_{n}\left(x\right)$. The upward recurrence of $\Psi_{n}\left(x\right)$ is initialized with $\Psi_{-1}\left(x\right)=\cos\left(x\right)$ and $\Psi_{0}\left(x\right)=\sin\left(x\right)$, and vice versa for the downward recurrence, while the upward recurrence of $\chi_{n}\left(x\right)$ starts with $\chi_{-1}\left(x\right)=\sin\left(x\right)$ and $\chi_{0}\left(x\right)=\cos\left(x\right)$, and vice versa for the downward recurrence [33].

### 4.6. Calculation of Extinction Coefficients

The extinction efficiencies $Q_{ext}$ and the drop-size distribution calculated in the previous section are used to calculate the extinction coefficients $\sigma_{ext,rain}$ and the backscattered coefficients $\beta_{back,rain}$. The extinction coefficients $\sigma_{ext,rain}$ can be written as:

(15) $$\sigma_{ext,rain}=\frac{\pi }{4}\int_{0}^{\infty }N\left(D\right)Q_{ext}D^{2}dD,$$

where N(D) is the rain distribution and *D* is the drop size diameter [29]. As mentioned earlier, we have used the rain distribution of Marshall–Palmer [24] and the NIED rain simulator [5].

### 4.7. Calculation of Backscattered Coefficients

A novel approach has been introduced to model the backscattering effect of rain droplets for the LiDAR sensor. First, the Monte Carlo Simulation generates all the rain droplets based on the DSD; then, a collision detection algorithm is applied between the transmitted ray of each scan point and the droplets. The collision detection algorithm determines the particles that collide with the virtual LiDAR ray by knowing the beam characteristics, such as beam volume, origin, and direction. These particles are described as a set of tuples:

(16) $$\mathrm{CP}=\{\left(R_{d},\theta_{az},\varphi_{el},D\right)\},$$

where $R_{d}$ is the distance of the particle from the sensor, the azimuth angle of the raindrop is denoted by $\theta_{az}$, and the elevation angle by $\varphi_{el}$, while *D* is the droplet diameter. The backscattered coefficient $\beta_{back,rain}$ is calculated for every droplet at the distance $R_{d}$.

(17) $$\beta_{back,rain}=\frac{\pi }{4}Q_{back}ND^{2}R_{d},$$

where $Q_{back}$ is the backscattered efficiency of the particle and *N* is the number of drops in 1/m${}^{3}$ [31].

### 4.8. Beam Characteristics

This work assumes a rectangular beam shape because the real LiDAR sensor used in this work has a rectangular beam shape; its near-field size can be described by the horizontal beam width $w_{ho}$ and the vertical beam width $w_{vo}$. With increasing range, the size of the beam also becomes larger in both directions. The divergence angles give this property with $\theta_{div}$ as the horizontal and $\varphi_{div}$ as the vertical divergence angle. For a given distance r>0 distance from the sensor, the size of the beam is [12]:

(18) $$w_{h}\left(r\right)=w_{ho}+r\;\cdot \;2tan\left(\frac{\theta_{div}}{2}\right)$$

and:

(19) $$w_{v}\left(r\right)=w_{vo}+r\;\cdot \;2tan\left(\frac{\varphi_{div}}{2}\right).$$

**Figure 5:**
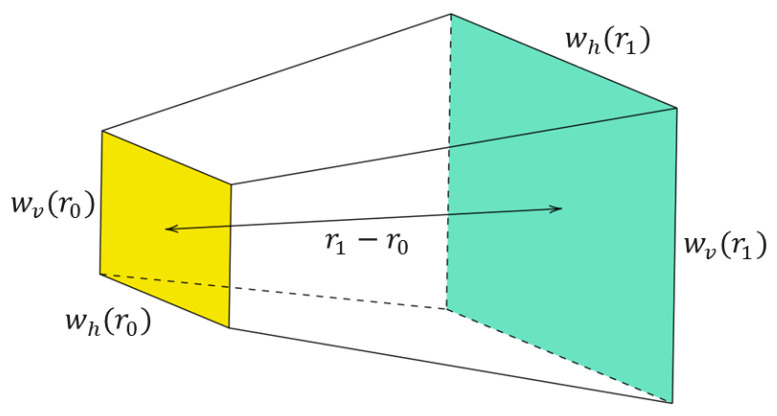
The geometry of a LiDAR ray from near-field to range $r_{1}$ considering beam divergence.

For the simulation of the effects of rain and fog, the volume of the beam between two given ranges, $r_{0}$ and $r_{1}$, is required (for instance, beam volume for range cells). As shown in Figure 5, it is trivial that the shape of the beam between $r_{0}$ and $r_{1}$ is a pyramidal frustum, the volume of which is given by:

(20) $$V=\frac{1}{3}h\;\cdot \;\left(A+\sqrt{\left(AB\right)}+B\right),$$

where *A* is the area of the top of the frustum marked in yellow, *B* is the base of the frustum shown in green, and *h* is the height of the frustum. Applying this formula to the beam itself results in:

(21) $$V_{r_{0},r_{1}}=\frac{1}{3}\left(r_{1}-r_{0}\right)\left(w_{h}\left(r_{0}\right)w_{v}\left(r_{0}\right)+\sqrt{w_{h}\left(r_{0}\right)w_{v}\left(r_{0}\right)w_{h}\left(r_{1}\right)w_{v}\left(r_{1}\right)}+w_{h}\left(r_{1}\right)w_{v}\left(r_{1}\right)\right).$$

This equation can be used to determine the volume of a beam in the field of view (FoV) or calculate the beam volume for each range cell depending on the range resolution.

### 4.9. Fog Module

There are two main approaches to model the effects of fog on the LiDAR signals: modeling via empirical formulas or modeling based on statistical distributions, as given in Section 4.2. All of these approaches have advantages and disadvantages. For instance, the LiDAR signal attenuation due to fog droplets can be modeled using empirical formulas introduced in [34,35,36], but it is not possible to model the backscattering from fog droplets. These approaches are easy to implement and computationally less expensive, while the statistical distributions that can be used to model the LiDAR signal attenuation and backscattering from the fog droplets are computationally expensive. In this work, we have therefore calculated the extinction coefficient $\sigma_{ext,fog}$ as a function of visibility distance *V* using the empirical formula recommended by the International Commission on Illumination (CIE) given in [9], which can be written as:

(22) $$\sigma_{ext,fog}=\frac{3}{V}.$$

The backscattering coefficient $\beta_{back,fog}$ is calculated using the Mie scattering theory and the Deirmendjian gamma distribution, and it can be written as:

(23) $$N\left(D\right)=\frac{\gamma \rho b^{\frac{\alpha +1}{\gamma }}}{\Gamma \left(\frac{\alpha +1}{\gamma }\right)}{\left(\frac{D}{2}\right)}^{\alpha }e^{\left(-b{\left(\frac{D}{2}\right)}^{\gamma }\right)},$$

where Γ(•) is the gamma function and α, γ, *b*, ρ, and *D* are the parameters of N(D),

(24) $$b=\frac{\alpha }{\gamma {\left(D_{C}/2\right)}^{\gamma }},$$

where $D_{c}$ denotes the mode diameter of maximum frequency droplets [9,12,37]. The distribution parameters for typical environmental conditions are given in Table 2.

The concentration of fog particles in the atmosphere is significantly higher than the number of rain particles, and their size *D* ranges from 0.1 μm to 20 μm [9,31]. The Monte Carlo Simulation approach adopted in Section 4.2 would be computationally highly expensive if used to generate virtual fog. Unlike the rain, we have therefore used an analytical method based on the Deirmendjian gamma distribution to generate homogenously distributed fog particles. We have used Equation (8) to calculate the backscattered efficiency $Q_{back}$. First, the virtual fog field is divided into range cells $R_{cells}$ based on the LiDAR range resolution ΔR to determine the backscattered coefficient $\beta_{back,fog}$. In the next step, we will consider all of the fog droplets within the beam volume of LiDAR as given in Equation (21) at any range cell $R_{cell}$ to determine the backscattered coefficient $\beta_{back,fog}$ from fog droplets. This can be written as:

(25) $$\beta_{back,fog}=R_{cell}\frac{\pi }{4}\int_{D=0}^{\infty }D^{2}N\left(D\right)Q_{back}dD,$$

where N(D) is the fog distribution, *D* denotes the drop diameter, and $Q_{back}$ is the backscattered efficiency [38].

### 4.10. Link Budget Module

The received power $P_{rx}\left(t\right)$ obtained from the ray tracing module does not consider the rain or fog effect and the receiver optics losses $T_{opt}$. The link budget considers the optical receiver losses $T_{opt}$ and attenuates the received power $P_{rx}\left(t\right)$ according to Beer–Lambert’s law using the rain or fog extinction coefficient $\sigma_{ext,rain/fog}$ obtained from the rain/fog module. This can be written as:

(26) $$P_{rx}\left(t\right)=\underset{P_{rx}\left(t\right)}{\underset{︸}{P_{tx}\left(t\right)\rho \frac{d_{aperture}^{2}}{4R_{trg}^{2}}cos\left(\theta \right)}}T_{opt}\;\cdot \;\exp\left(-2R_{trg}\sigma_{ext,rain/fog}\right),$$

where ρ is the target reflectivity, $d_{aperture}$ denotes the diameter of the optical aperture, $R_{trg}$ is the target range, the direction of the incident ray is given by θ, the receiver optics loss factor is given by $T_{opt}$, and the transmitted power is denoted by $P_{tx}\left(t\right)$ [4,12]. The backscattered coefficient $\beta_{back}$ is used to calculate the received power $P_{rx}$ of backscattered rain or fog droplets. The received power $P_{rx}$ from the raindrops can be written as:

(27) $$P_{rx,\;back\;rain}\left(t\right)=P_{tx}\left(t\right)\frac{d_{aperture}^{2}}{4R_{d}^{2}}cos\left(\theta \right)T_{opt}\;\beta_{back,\;rain}\;\cdot \;\exp\left(-2R_{d}\sigma_{ext,rain}\right),$$

where $\beta_{back,\;rain}$ is the backscattered coefficient from raindrops and $R_{d}$ is the raindrop range [4,12]. The received power $P_{rx,\;back\;fog}\left(t\right)$ from the fog droplets in a range cell can be written as:

(28) $$P_{rx,\;back\;fog}\left(t\right)=P_{tx}\left(t\right)\frac{d_{aperture}^{2}}{4R_{cell}^{2}}cos\left(\theta \right)T_{opt}\;\beta_{back,\;fog}\;\cdot \;\exp\left(-2R_{cell}\sigma_{ext,fog}\right),$$

where $\beta_{back,\;fog}$ is the backscattered coefficient from the fog droplets in any range cell and $R_{cell}$ is the distance of the range cell.

The total $P_{tot}\left(t\right)$ received power by the detector over time can originate from different sources, including internal reflection $P_{int}\left(t\right)$, target received power $P_{rx}\left(t\right)$, and backscattered power from rain and fog drops $P_{rx,\;rain/\;fog}$. That is why $P_{tot}\left(t\right)$ can be given as:

(29) $$P_{tot}\left(t\right)=P_{int}\left(t\right)+P_{rx}\left(t\right)+P_{rx,\;rain/\;fog}\left(t\right).$$

The power signal must be sampled with a Δt time interval to accurately model the optics at the photon level [39]. The sampled power equation takes the form of:

(30) $$P_{tot}\left[i\right]=P_{int}\left[i\right]+P_{rx}\left[i\right]+P_{rx,\;rain/fog}\left[i\right],$$

with t=i·Δt. The mean of incident photons $\bar{n}\left[i\right]$ on the SiPM detector within a one-time bin can be written as:

(31) $$\bar{n}\left[i\right]=\frac{P_{tot}\left[i\right]\;\cdot \;\Delta t}{E_{ph}},$$

where $E_{ph}=h\nu$ is the energy of a single laser photon at the laser’s wavelength, *h* is the Planck constant, and ν is the photon frequency [40]. The SiPM detector generates Poisson-distributed shot noise due to the statistical arrival of photons. That is why the arrival of photons can be modeled as a Poisson process P [41]:

(32) $$n\left[i\right]=P(\bar{n}\left[i\right]).$$

### 4.11. Detector Module

We have implemented the SiPM (silicon photomultiplier) detector module that provides an output current proportional to the number of photons [39]. In contrast to the single-photon avalanche diode (SPAD), the SiPM detector yields better multi-photon detection sensitivity, photon number resolution, and extended dynamic range [42,43]. The SiPM detector response for a given photon signal can be calculated as:

(33) $$i_{d}\left[i\right]=S_{i}\;\cdot \;\left(h_{SiPM}\left[i\right]*n\left[i\right]\right),$$

where $S_{i}$ is the SiPM detector sensitivity and the impulse response of the detector, written as $h_{SiPM}$. $S_{i}$, is given as:

(34) $$S_{i}=1-e^{\left(-t/\tau_{delay}\right)},$$

where $\tau_{delay}$ is the SiPM recovery time [39,42,43].

### 4.12. Circuit Module

We use the small-signal transfer function H(f) of the analog circuit model to obtain the voltage signal $v_{c}\left[i\right]$,

(35) $$v_{c}\left[i\right]=v_{c0}+\Delta v_{c}\left[i\right]=v_{c0}+F^{-1}\left\{H\left(f\right)\;\cdot \;\underset{F\{i_{d}\left[i\right]\}}{\underset{︸}{I_{d}\left(f\right)}}\right\},$$

where $v_{c0}$ is the operating voltage of the circuit model, $F^{-1}$ is the inverse discrete Fourier transform (IDFT), F shows the discrete Fourier transform (DFT), and $\Delta v_{c}\left[i\right]$ denotes the small-signal voltage of the circuit model [39].

### 4.13. Ranging Module

The ranging algorithm inputs the circuit module’s voltage signal $v_{c}\left[i\right]$. It then calculates each scan point’s target range *R* and signal intensity *I*. The range *R* is given in meters while the intensity *I* is mapped linearly to an arbitrary integer scale from 0 to 4096 as used in the Cube 1 products. The algorithm is applied to several threshold levels to distinguish between internal reflection, noise, and target peaks. The target range is determined based on the relative position of the target peaks to the internal reflection, while the signal intensity is calculated from the peak voltage levels [4].

## 5. Results

The rain and fog effect modeling is validated on the time domain and point cloud levels. As shown in the following, we have used a single-point scatter to validate the model on the time domain.

### 5.1. Validation of the Rain Effect Modeling on the Time Domain Level

**Figure 6:**
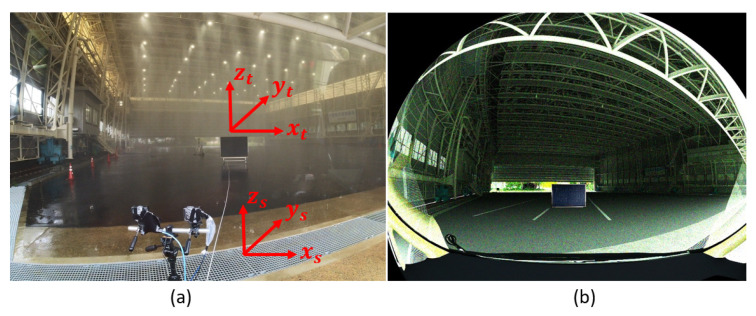
(**a**) Real setup to validate the time domain and point cloud data. (**b**) Static simulation scene to validate the time domain and point cloud data. The 3%-reflective target with an area of 1.3 m × 1.3 m was placed in front of the sensor at different distances. The actual and the simulated sensor and target coordinates are the same. The ground truth distance $d_{GT}$ is calculated from the sensor’s origin to the target’s center.

The primary reason for verifying the LiDAR model on the time domain is to ensure that the link budget, detector, and circuit modules work as intended. Furthermore, comparing the time domain signals (TDS) establishes the association between measured and modeled noise and the amplitude levels because it is difficult to compare the simulated and measured noise at the point cloud level. It is, therefore, convenient to quantify the LiDAR signal attenuation $\sigma_{ext}$ and the decrease in the SNR due to the rain and fog droplets on the time domain level. A 3%- and a 10%-reflective Lambertian plate were placed in front of the sensor at 20 m in the rain rate $r_{rate}$ of 16 mm/h, 32 mm/h, 66 mm/h, and 98 mm/h. An exemplary scenario for a 3% Lambertian plate is shown in Figure 6.

**Figure 7:**
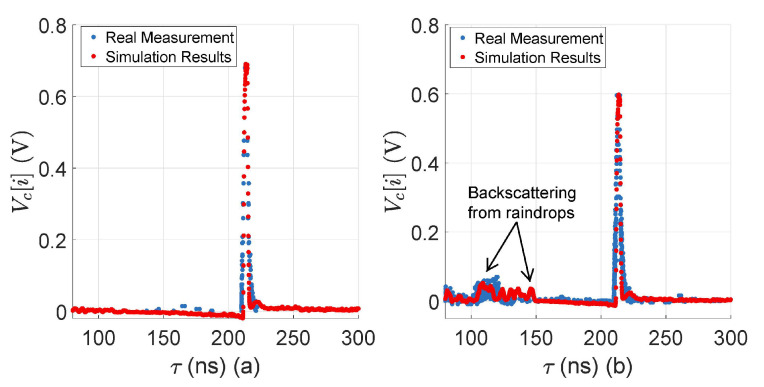
(**a**) LiDAR FMU and real measured TDS comparison obtained from the surface of a 10%-reflective Lambertian plate at 20 m without rain. The target peaks and noise levels match well. (**b**) LiDAR FMU and real measured TDS comparison obtained from the surface of a 10%-reflective Lambertian plate placed at 20 m with 16 mm/h rain rate $r_{rate}$. The target peaks and the amplitude level of the backscattered raindrops match well. It should be noted that the relative distance is calculated from the internal reflection to the target peaks.

Both the simulated and the real measured TDS obtained from the 10%-reflective plate are shown in Figure 7, both with and without rain at 16 mm/h. There is a good match between the target peaks and the amplitude level of the backscattered raindrops, but still there is a slight difference in the amplitude level of the backscattered raindrops because real-world rainfall is a random process, and it is impossible to replicate the exact behavior of real-world rain in the simulation, yet the simulation and the real measurements agree well with each other.

**Figure 8:**
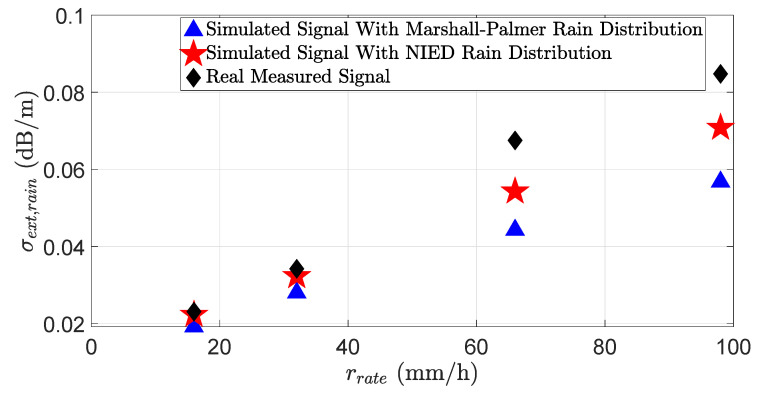
Simulated and real LiDAR signals attenuation $\sigma_{ext,rain}$ due to different rain rates $r_{rate}$. The simulation results with Marshall–Palmer, NIED rain distribution, and real measurements match very well at lower rain rates. However, as the rain rate increases, the simulation and real measurement signal attenuation mismatch also increases, especially for the simulation results with the Marshall–Palmer rain distribution.

**Figure 9:**
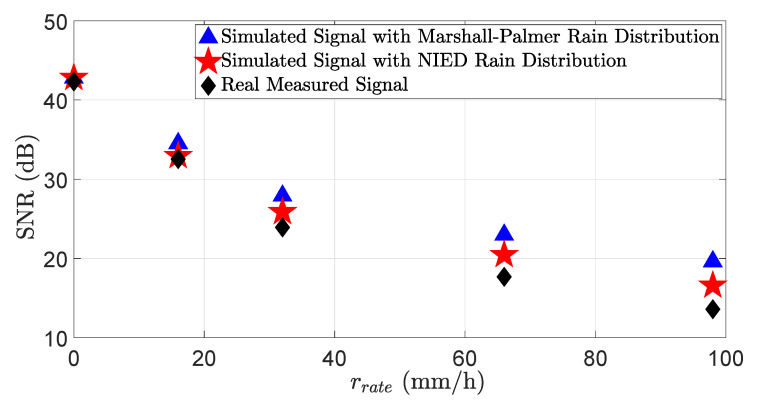
The SNR of the simulated and real measured signals in different rain rates $r_{rate}$. The simulation and real measurement results match very well at lower rain rates, but the mismatch between the simulated and real SNR increases as the rain rate increases, especially for the simulation results with the Marshall–Palmer rain distribution.

Figure 8 shows the LiDAR signal attenuation $\sigma_{ext,rain}$ at different rain rates $r_{rates}$. The results show that the LiDAR signal attenuation significantly depends on the rain distribution. Therefore, the simulation and real measurements will match well if the simulated rain distribution is close to the real distribution. Figure 9 shows the SNR of the simulated and real measured signals in different rain rates $r_{rate}$. The SNR can be calculated as:

(36) $$SNR=20\;log_{10}\left(\frac{\mu }{\sigma }\right),$$

where μ is the mean or expected signal value and σ is the standard deviation of noise [44]. The result shows that at the lower rain rates $r_{rate}$, the SNR of the simulated and measured signals match well, but just as the rain rate $r_{rate}$ increases, the SNR mismatch also increases, especially for the simulation results with the Marshall–Palmer rain distribution. Therefore, the simulation and real measurements for SNR will also match well if the simulated rain distribution is close to the real distribution. The backscattering from the raindrops is a random process, and it is not always possible to generate the same backscattering amplitude from the virtual raindrops as from real-world raindrops, and this is also a reason for the mismatch between the real and simulated LiDAR signals’ SNR.

To quantify the difference between the simulated and real measured signals’ attenuation $\sigma_{ext,rain}$ and SNR, we use the mean absolute percentage error (MAPE) metric:

(37) $$MAPE=\frac{1}{n}\sum_{i=1}^{n}|\frac{y_{i}-x_{i}}{y_{i}}|,$$

where $y_{i}$ is the measured value, the simulated value is denoted by $x_{i}$, and *n* shows the total number of data points [45]. The MAPE of the signal attenuation $\sigma_{ext}$ for the NIED rain distribution is 16.2% and 31.6% for the Marshall–Palmer rain distribution model. Moreover, the MAPE of the SNR for the NIED rain distribution is 4.3% and 9.1% for the Marshall–Palmer rain distribution model. The results show that the LiDAR sensor’s behavior will vary significantly depending on the rain distribution.

### 5.2. Validation of the Rain Effect Modeling on the Point Cloud Level

We have introduced three KPIs to validate the rain effect modeling on the point cloud level: the LiDAR detecting rate (DR), the false detection rate (FDR), and the distance error $d_{error}$.

1. The DR is defined as the ratio between the number of returns obtained from both real and simulated objects of interest (OOI) in rainy $\left(\#\;returns\;OOI_{rain,\;sim/real}\right)$ and dry $\left(\#\;returns\;OOI_{dry,\;sim/real}\right)$ conditions. It can be written as:
  (38) $$DR=\frac{\#\;returns\;OOI_{rain,\;sim/real}}{\#\;returns\;OOI_{dry,\;sim/real}}.$$
  It should be noted that the number of points obtained from OOI in rainy and dry conditions are the mean over all measurements of the same scenario.
2. The FDR of the LiDAR sensor in rainy conditions can be written as:
  (39) $$FDR=\frac{\#\;returns_{rain,\;sim/real}-\#\;returns\;OOI_{rain,\;sim/real}}{\#\;returns\;OOI_{dry,\;sim/real}},$$
  where $\#\;returns_{rain,\;sim/real}$ is the total number of reflections from the sensor minimum detection range to the simulated and real OOI, and it does not contain any reflection from the OOI’s surroundings. $\#\;returns\;OOI_{rain,\;sim/real}$ and $\#\;returns\;OOI_{dry,\;sim/real}$ depict the LiDAR returns from the surface of the simulated and real OOI in rainy and dry conditions. It should be noted that the number of LiDAR reflections obtained under rainy and dry conditions are the mean over all measurements of the same scenario.
3. The distance error $d_{error}$ of the point cloud received from OOI in rainy and dry conditions, both simulated and real, can be written as:
  (40) $$d_{error}=d_{GT}-d_{mean,\;dry/rain,\;sim/real},$$
  where the ground truth distance is denoted by $d_{GT}$ and $d_{mean,\;dry/rain,\;sim/real}$ is the mean distance of reflections received from the surface of the simulated and the real OOI in rainy and dry conditions. The ground truth distance $d_{GT}$ is calculated from the sensor’s origin to the target’s center, and it can be written as:
  (41) $$d_{GT}=\sqrt{{\left(x_{t}-xs\right)}^{2}+{\left(y_{t}-y_{s}\right)}^{2}+{\left(z_{t}-z_{s}\right)}^{2}},$$
  where the target’s *x*, *y*, and *z* coordinates are denoted by subscript *t* and the sensors by *s* [46]. The OSI ground truth interface *osi3::GroundTruth* is used to retrieve the sensor origin and target center position in 3D coordinates.

Table 3 gives the DR of the real and virtual LiDAR sensor in different rain conditions for a 3%-reflective Lambertian plate. The results show that up to 20 m, the LiDAR sensor can reliably detect a very low-reflective target with a rain rate of 98 mm/h. It should be noted that these results were obtained in a rain facility area where the rain was homogenously distributed. For the benchmarking of the DR, we have filtered the point clouds from the edges of the Lambertian plate, meaning that the effective area of the plate becomes 1.1 m × 1.1 m. The simulation and the real measurements show a good correlation with each other. The MAPE for the DR is 2.1%.

**Figure 10:**
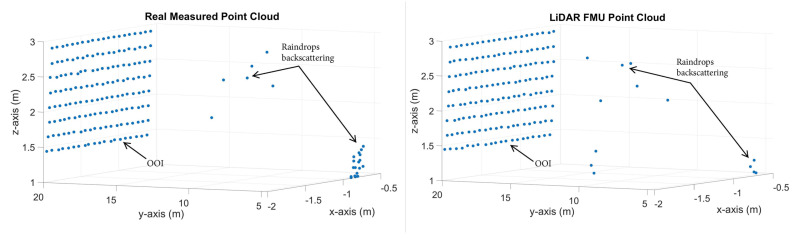
The exemplary visualization of simulated and real point clouds obtained in 32 mm/h rain rate $r_{rate}$.

Table 4 shows the FDR of the real and the virtual LiDAR sensor in different rain conditions for a 3%-reflective Lambertian plate. It should be noted that the FDR are the false positive detections from the raindrops. The results show that while the rain rate and the relative distance between the sensor and the target increase, the FDR also increases because as the rain rate increases, the size of the raindrops also increases, leading to a higher backscattering of LiDAR rays from the raindrops, which ultimately results in more FDR. It should be noted that the FDR also depends on the rain distribution and sensor mounting position. Furthermore, the simulation and the real measurements show good agreement. The MAPE for the FDR is about 14.7%. The exemplary point clouds obtained from the surface of the real and the simulated 3%-reflective Lambertian plate at a rain rate of 32 mm/h are shown in Figure 10.

Table 5 gives the distance error $d_{error}$ of both real and virtual LiDAR sensors in different rain conditions. It shows that the distance error $d_{error}$ increases with the increase in the rain rate $r_{rate}$ because as the rain rate increases, the size of the raindrops also grows. Once the LiDAR rays collide with them, the drops cause the LiDAR rays to misalign; that is why the distance error increases for higher rain rates.

### 5.3. Validation of the Fog Effect Modeling on the Time Domain Level

**Figure 11:**
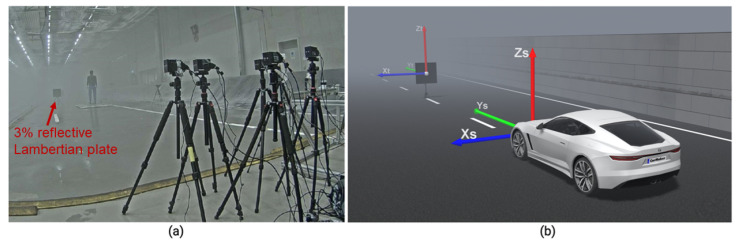
(**a**) The real setup for the fog measurement. (**b**) The static simulation scene for the validation of the fog effect. The 3%-reflective Lambertian plate was placed at a 15.3 m distance. The real and virtual LiDAR sensor and targets coordinates are the same.

**Figure 12:**
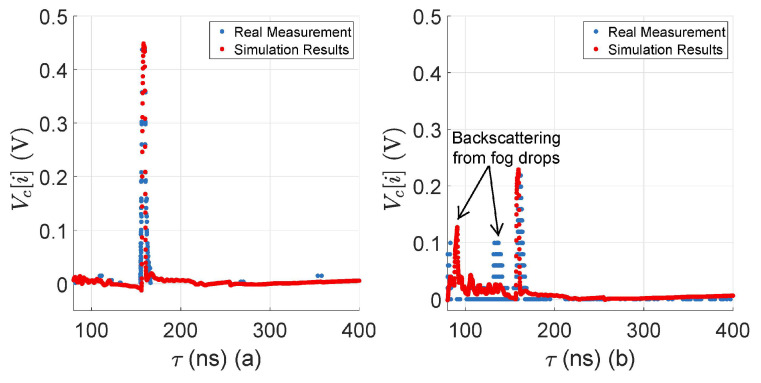
(**a**) LiDAR FMU and real measured TDS obtained from the surface of a 3%-reflective plate placed at 15.3 m without fog. The target peaks and noise levels match well. (**b**) LiDAR FMU and real measured TDS obtained from the surface of a 3%-reflective plate at 15.3 m with a fog visibility *V* of 140 m. It should be noted that the relative distance is calculated from the internal reflection to the target peaks.

To verify the modeling of the fog effect, we have placed the 3%-reflective Lambertian plate at 15.3 m in front of the sensor, as shown in Figure 11. Both the simulated and the real measured signals that reflect from the surface of the 3%-reflective Lambertian plate, with and without fog, are shown in Figure 12.

**Figure 13:**
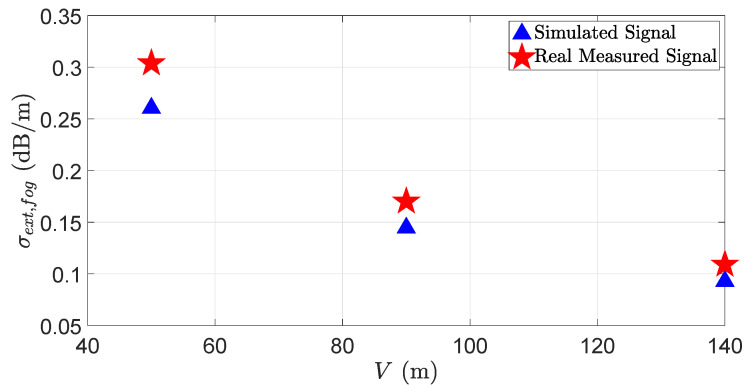
The real and virtual LiDAR signals attenuation $\sigma_{ext,fog}$ due to the different visibility distances *V*. The simulation and real measurement results match well at the higher visibility distances. However, as the visibility distance decreases due to fog, the simulated and real measured signal attenuation mismatch increases.

**Figure 14:**
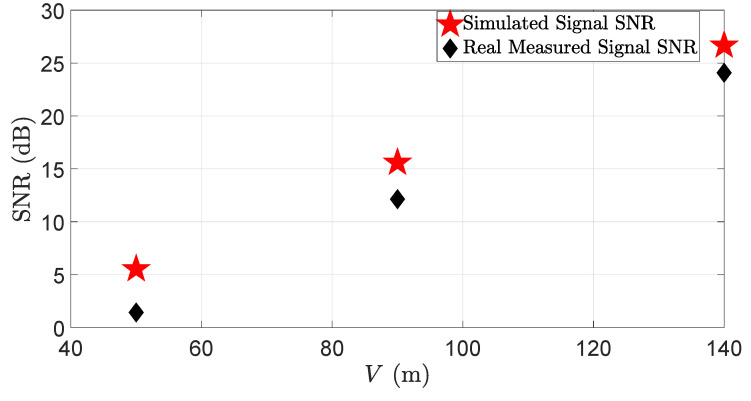
The SNR of the simulated and real measured signals with different visibility distances *V*. The simulation and real measurement results match well at the higher visibility distances, but the mismatch between the simulated and real measured SNR increases as the visibility distance decreases.

The results show that the simulated and the real measured target peaks and noise levels match well without and with fog. The peak location of backscattering from the fog droplets in the simulation and the real measurement varies because it is a random process, and replicating the exact real-world fog behavior in the simulation is challenging. For instance, the fog drop size changes during the fog life cycle [47,48], affecting the LiDAR sensor performance. Measuring and replicating the change in the fog droplet size during the fog life cycle in the virtual environment is quite challenging. Moreover, in a virtual environment, it is easy to maintain constant visibility to a certain distance. It was a challenge, however, to retain constant visibility to a certain distance, both in the real world and in laboratory-controlled conditions, due to the limitations of the measurement instruments and the fog generation setup. Figure 13 and Figure 14 show the simulated and the real LiDAR signal attenuation $\sigma_{ext,fog}$ and SNR with different visibility distances *V*. The result shows that the LiDAR signal attenuation $\sigma_{ext,fog}$ caused by fog droplets increases while the visibility distances *V* decreases; for instance, the LiDAR signal attenuation increases to 9.2 dB with a visibility distance of 50 m. The result also shows that the dense fog increases the mismatch of the simulated and the real measured signals for the attenuation and SNR. As mentioned above, the possible reason behind these deviations is the non-constant distribution of fog in the measurement area and the random backscattering process from fog droplets.

We use the MAPE metric as given in Equation (37) to quantify the difference between the simulated and the real measured results for fog. The MAPE for the signal attenuation due to fog $\sigma_{ext,fog}$ is 13.9% and 15.7% for SNR.

### 5.4. Validation of the Fog Effect Modeling on the Point Cloud Level

**Figure 15:**
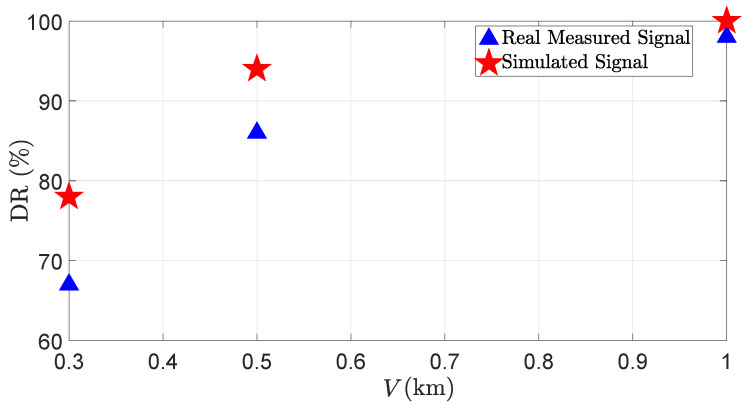
The DR of the LiDAR sensor for the real and virtual 3%-reflective Lambertian plate with different visibility distances *V*. The simulation and real measurements show good correlations.

To validate the fog effect modeling on the point cloud level, we consider the same KPIs as given in Section 5.2. Figure 15 shows the DR of the real and the virtual LiDAR sensor for different visibility distances *V*.

**Figure 16:**
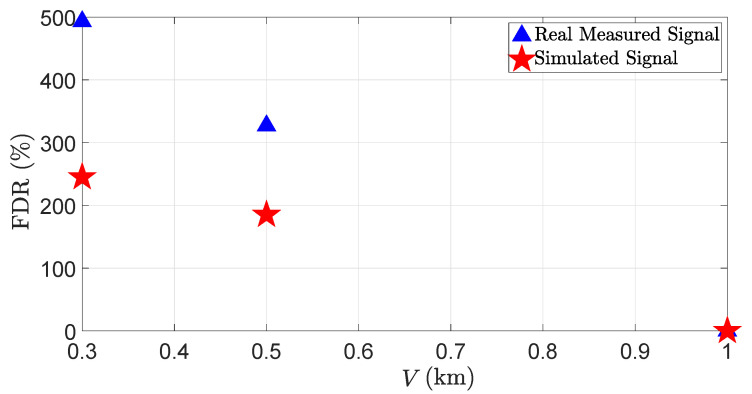
The FDR of the LiDAR sensor for a real and virtual 3%-reflective Lambertian plate with different visibility distances *V*. The FDR increases with a decrease in the visibility distances. It should be noted that FDR 300% or 500% shows that the LiDAR reflections received from the fog droplets are 3 or 5 times more than those obtained from the OOI (see Equation (39)).

It shows that as the visibility distances decreases, the DR of the real and the virtual LiDAR sensors also decreases. The size of the fog droplets ranges from 5 to 20 μm, and 1 m^3^ of air contains $10^{6}$ fog droplets, which is $10^{3}$ times more than the number of raindrops in 1 m^3^ of air [31]. Therefore, it is easier for LiDAR rays to penetrate through raindrops suspended in the air than fog droplets. That is why the LiDAR signal attenuation is higher in dense fog than in heavy rain. Furthermore, the simulation and the real measurements show a good correlation. The MAPE for the DR is 7.5%. Figure 16 shows the FDR of the real and the virtual LiDAR sensor due to fog.

The results show that as the visibility distance *V* decreases, the FDR of the real and virtual LiDAR sensors increases. The results also show that the mismatch between the simulation and the real measurements for the FDR increases at low visibility distances. It should be noted that the backscattering from the fog droplets is a random process, and it is, therefore, impossible to model the exact behavior of real-world fog in the simulation. Furthermore, we have not measured nor modeled the real fog distribution due to limited resources. This could be another reason for the mismatch between the simulation and the real measurements. The MAPE for the FDR is 48.2%.

**Figure 17:**
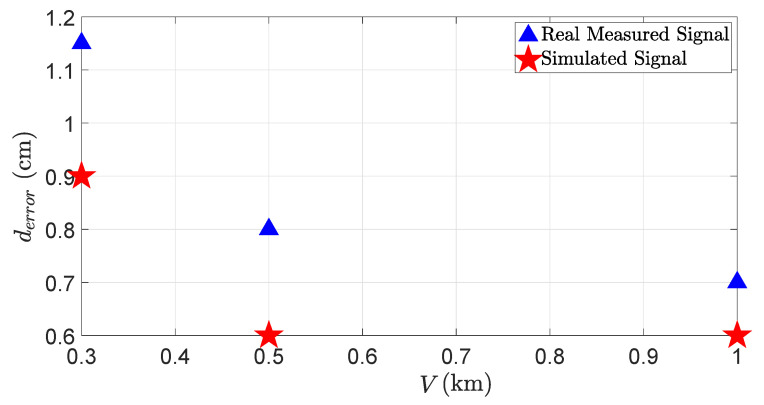
The distance error $d_{error}$ of the LiDAR sensor for the real and virtual 3%-reflective Lambertian plate with different visibility distances *V*. The distance error $d_{error}$ increases with the decrease in visibility distances. The simulation and real measurements show a good correlation.

Figure 17 shows the distance error $d_{error}$ due to fog droplets. The results show that the distance error also increases as the visibility distance *V* decreases, but still, the distance error does not exceed the maximum permissible error (MPE) specified by the manufacturer, which is 2 cm in this case. Because the size of fog droplets is several magnitudes smaller than the size of raindrops, LiDAR rays are less misaligned by the scattering from the fog droplets than raindrops. That is why the distance error due to the fog droplets is smaller than the one caused by raindrops.

## 6. Conclusions

In this work, we introduce a novel approach to model the rain and fog effect in the virtual LiDAR sensor with high fidelity. The presented approach allows for the rain and fog effect simulation in the LiDAR model on the time domain and point cloud level. Furthermore, validating the LiDAR sensor model on the time domain enabled us to benchmark the LiDAR signal attenuation $\sigma_{ext}$ and SNR. The results show that the virtual and real LiDAR sensor signal attenuation $\sigma_{ext,rain}$ and SNR match well if both simulated and real rain distributions are the same; for instance, the MAPE of the signal attenuation for the NIED rain distribution is 16.2%, while the MAPE for the Marshall–Palmer rain distribution is 31.6%. In addition, the MAPE of the SNR for the NIED rain distribution is 4.3% and 9.1% for the Marshall–Palmer rain distribution model. Therefore, the simulation and real measurement results will show a good correlation if the simulated and real rain distribution are the same.

The results also show an increase in the LiDAR signal attenuation $\sigma_{ext,fog}$ caused by fog droplets, while the visibility distance *V* decreases; for instance, the LiDAR signal attenuation increases to 9.2 dB with a visibility distance of 50 m. Furthermore, the simulation and the real measurements show good agreement for the signal attenuation and SNR due to fog droplets. The MAPE for the signal attenuation is about 13.9% and 15.7% for SNR due to fog droplets.

To validate the modeling of the effect of rain and fog at the point cloud level, we have introduced three KPIs: DR, FDR, and distance error $d_{error}$. The results show that with the increasing rain rate $r_{rate}$, DR decreases, while both the real and virtual LiDAR sensors could detect the 3%-reflective target at 20 m with a rain rate of 98 mm/h. On the other hand, both FDR and distance error increase with an increase in the rain rate. Moreover, the simulation and the real measurements show a good correlation; for instance, the MAPE for DR is 2.1%, and the MAPE for FDR is 14.7%.

DR significantly decreases in fog with a decrease in the visibility distance because the density of fog droplets is $10^{3}$ times higher than the density of raindrops in 1 m^3^ of air. As a consequence, LiDAR rays cannot easily penetrate through fog. FDR also increases with the decrease in visibility distances *V*, but the distance error is less than 2 cm. As a fog droplet is several magnitudes smaller than a raindrop, LiDAR rays become less misaligned by the scattering from fog droplets as opposed to the scattering from raindrops. However, the simulation and the real measurements correlate well for these KPIs. For example, the MAPE for DR is 7.5%, while MAPE for FDR is 48.2%. It should be noted that the backscattering from the fog droplets is a random process, and it is, therefore, impossible to model the exact behavior of real-world fog in the simulation. Furthermore, we have not measured nor modeled the real fog distribution due to limited resources. This could be another reason for the mismatch between the simulation and the real measurements. For the LiDAR sensor, it is easier to detect the low-reflective target easily in heavy rain, but it is very challenging for the LiDAR sensor to detect the target reliably once the visibility distance drops below 100 m.

## 7. Outlook

In the next step, we will train the deep learning network-based LiDAR detector with the data from the simulated rain and fog effect to improve object recognition in rainy and foggy situations.

## Figures and Tables

**Table 1:** Overview of the state-of-the-art LiDAR sensor model working principles and validation approaches.

Authors	Covered Weather Phenomena	Covered Effects	Validation Approach
Goodin et al. [7]	Rain	Signal attenuation, false negative, ranging error $d_{error}$, decrease in maximum detection range	Simulation results
Wojtanowski et al. [8]	Rain, fog, aerosols	Signal attenuation, target reflectivity, range degradation	Simulation results
Rasshofer et al. [9]	Rain, fog, snow	Signal attenuation, range degradation	Simulation results, qualitative comparison with real measurements for fog attenuation
Byeon et al. [10]	Rain	Signal attenuation	Simulation results
Li et al. [11]	Rain, fog, snow, haze	Signal attenuation	Simulation results
Zhao et al. [12]	Rain, fog, snow, haze	Signal attenuation, false positive	Quantitative comparison with measurements for rain
Guo et al. [13]	Rain	Signal attenuation	Qualitative comparison with measurements
Hasirlioglu et al. [14,15]	Rain	Signal attenuation, false positive	Quantitative comparison with measurements
Berk et al. [16]	Rain	Signal attenuation, false positive	Simulation results
Espineira et al. [17]	Rain	Signal attenuation, false positive	Simulation results
Kilic et al. [18]	Rain, fog, snow	Signal attenuation, false positive	Quantitative comparison with measurements
Hahner et al. [19]	Fog	Signal attenuation, false positive	Quantitative comparison with measurements
Haider et al. (proposed approach)	Rain, fog	Signal attenuation, SNR, false positive, false negative, ranging error $d_{error}$	Qualitative comparison with measurements for all covered effects

**Table 2:** Parameters of droplet size distribution in fog using a gamma-function model [9].

Weather Condition	ρ (${\mathrm{cm}}^{-3}$)	α	γ	$D_{C}\;\left(\mu m\right)$
Haze (coast)	100	1	0.5	0.1
Haze (continental)	100	2	0.5	0.14
Strong advection fog	20	3	1.0	20.0
Moderate advection fog	20	3	1.0	16.0
Strong spray	100	6	1.0	8.00
Moderate spray	100	6	1.0	4.00
Fog of type “Chu/Hogg”	20	2	0.5	2.00

**Table 3:** The DR of the LiDAR sensor, both for a real and a virtual 3%-reflective Lambertian plate, without rain and with different rain rates $r_{rate}$. The simulation and real measurements show a good correlation. The table presents the mean over 154 measurements of the same scenario. The real LiDAR sensor DR is denoted by $DR_{real}$, $DR_{sim}$ is the virtual LiDAR sensor DR, and $\Delta DR=|DR_{real}-DR_{sim}|$.

$r_{rate}$ (mm/h)					Target Distance *R*							
	5 m			10 m			15 m			20 m		
	$DR_{real}$ (%)	$DR_{sim}$ (%)	ΔDR (%)	$DR_{real}$ (%)	$DR_{sim}$ (%)	ΔDR (%)	$DR_{real}$ (%)	$DR_{sim}$ (%)	ΔDR (%)	$DR_{real}$ (%)	$DR_{sim}$ (%)	ΔDR (%)
0	100.0	100.0	0.0	100.0	100.0	0.0	100.0	100.0	0.0	100.0	100.0	0.0
16	100.0	100.0	0.0	100.0	100.0	0.0	89.3	96.7	7.4	88.1	93.9	5.8
32	100.0	100.0	0.0	100.0	100.0	0.0	87.5	93.2	5.7	85.3	88.4	3.1
66	100.0	100.0	0.0	99.8	100.0	0.2	86.2	91.6	5.4	84.4	89.2	4.8
98	100.0	100.0	0.0	96.5	100.0	3.5	85.2	88.2	3.0	82.3	85.9	3.6

**Table 4:** The FDR of the LiDAR sensor, both for a real and a virtual 3%-reflective Lambertian plate, for different rain rates $r_{rate}$. The simulation and real measurements show a good correlation. The graph presents the mean over 154 measurements of the same scenario. The real LiDAR sensor FDR is denoted by $FDR_{real}$, $FDR_{sim}$ is the virtual LiDAR sensor FDR, and $\Delta FDR=|FDR_{real}-FDR_{sim}|$.

$r_{rate}$ (mm/h)					Target Distance *R*							
	5 m			10 m			15 m			20 m		
	$FDR_{real}$ (%)	$FDR_{sim}$ (%)	ΔFDR (%)	$FDR_{real}$ (%)	$FDR_{sim}$ (%)	ΔFDR (%)	$FDR_{real}$ (%)	$FDR_{sim}$ (%)	ΔFDR (%)	$FDR_{real}$ (%)	$FDR_{sim}$ (%)	ΔFDR (%)
0	0.0	0.0	0.0	0.0	0.0	0.0	0.0	0.0	0.0	0.0	0.0	0.0
16	0.8	0.3	0.5	1.8	1.4	0.4	3.2	2.9	0.3	5.5	4.1	1.4
32	1.6	1.0	1.6	4.8	3.1	1.7	7.1	5.8	1.4	7.3	7.6	0.3
66	1.7	1.2	0.5	7.0	5.7	1.3	18.9	14.6	4.3	19.6	18.2	1.4
98	2.4	1.9	0.5	9.1	6.9	2.2	20.4	17.2	3.2	22.7	20.2	2.5

**Table 5:** The distance error $d_{error}$ of the LiDAR sensor, both for a real and a virtual 3%-reflective Lambertian plate, in different rain rates $r_{rate}$. The simulation and real measurements show a good correlation. The graph presents the mean over 154 measurements of the same scenario. The real LiDAR sensor distance error is denoted by $d_{error,real}$, the virtual LiDAR sensor distance error is given by $d_{error,sim}$, and $\Delta d_{error}=\left|d_{error,real}-d_{error,sim}\right|$.

$r_{rate}$ (mm/h)					Target Distance *R*							
	5 m			10 m			15 m			20 m		
	$d_{error,real}$ (cm)	$d_{error,sim}$ (cm)	$\Delta d_{error}$ (cm)	$d_{error,real}$ (cm)	$d_{error,sim}$ (cm)	$\Delta d_{error}$ (cm)	$d_{error,real}$ (cm)	$d_{error,sim}$ (cm)	$\Delta d_{error}$ (cm)	$d_{error,real}$ (cm)	$d_{error,sim}$ (cm)	$\Delta d_{error}$ (cm)
0	0.2	0.1	0.1	0.5	0.1	0.4	0.7	0.2	0.5	1.4	0.2	1.2
16	1.1	0.9	0.2	1.3	1.1	0.2	1.7	1.4	0.3	2.3	1.5	0.8
32	1.2	1.0	0.2	1.8	1.2	0.6	2.9	2.0	0.9	3.3	2.2	1.1
66	1.4	1.1	0.3	2.6	1.9	0.7	3.0	2.2	0.8	4.8	2.4	2.4
98	1.6	1.2	0.4	2.9	1.6	1.3	3.1	2.3	0.9	4.9	2.8	2.1

## Data Availability

Not applicable.

## Abbreviations

The following abbreviations are used in this manuscript:

ADAS	Advanced driver-assistance system
BRDF	Bidirectional reflectance distribution function
CIE	International Commission on Illumination
DFT	Discrete Fourier transform
DR	Detection rate
DSD	Drop size distribution
FDR	False detection rate
FMU	Functional mock-up unit
FMI	Functional mock-up interface
FoV	Field of view
IDFT	Inverse discrete Fourier transform
KPIs	Key performance indicators
MAPE	Mean absolute percentage error
MPE	Maximum permissible error
NIED	National Research Institute for Earth Science and Disaster Prevention
OSI	Open simulation interface
OOI	Object of interest
RADAR	Radio detection and ranging
RTDT	Round-trip delay time
SNR	Signal-to-noise ratio
TDS	Time domain signals
